# Single-cell RNA sequencing analysis of the temporomandibular joint condyle in 3 and 4-month-old human embryos

**DOI:** 10.1186/s13578-023-01069-5

**Published:** 2023-07-19

**Authors:** Qianqi Zhu, Miaoying Tan, Chengniu Wang, Yufei Chen, Chenfei Wang, Junqi Zhang, Yijun Gu, Yuqi Guo, Jianpeng Han, Lei Li, Rongrong Jiang, Xudong Fan, Huimin Xie, Liang Wang, Zhifeng Gu, Dong Liu, Jianwu Shi, Xingmei Feng

**Affiliations:** 1 Department of Stomatology, Affiliated Hospital of Nantong University, Medical School of, Nantong University, Nantong, 226001 China; 2 Research Center of Clinical Medicine, Affiliated Hospital of Nantong University, Medical School of, Nantong University, Nantong, 226001 China; 3 Institute of Reproductive Medicine, Medical School of Nantong University, Nantong, 226001 China; 4 School of Life Science, Nantong Laboratory of Development and Diseases Second Affiliated Hospital Key Laboratory of Neuroregeneration of Jiangsu and Ministry of Education, Co-Innovation Center of Neuroregeneration, Nantong University, Nantong, 226019 China

**Keywords:** TMJC, Human embryonic cells, Cellular transcriptomics, Gene expression

## Abstract

**Background:**

The temporomandibular joint (TMJ) is a complex joint consisting of the condyle, the temporal articular surface, and the articular disc. Functions such as mastication, swallowing and articulation are accomplished by the movements of the TMJ. To date, the TMJ has been studied more extensively, but the types of TMJ cells, their differentiation, and their interrelationship during growth and development are still unclear and the study of the TMJ is limited. The aim of this study was to establish a molecular cellular atlas of the human embryonic temporomandibular joint condyle (TMJC) by single-cell RNA sequencing, which will contribute to understanding and solving clinical problems.

**Results:**

Human embryos at 3 and 4 months of age are an important stage of TMJC development. We performed a comprehensive transcriptome analysis of TMJC tissue from human embryos at 3 and 4 months of age using single-cell RNA sequencing. A total of 16,624 cells were captured and the gene expression profiles of 15 cell clusters in human embryonic TMJC were determined, including 14 known cell types and one previously unknown cell type, "transition state cells (TSCs)". Immunofluorescence assays confirmed that TSCs are not the same cell cluster as mesenchymal stem cells (MSCs). Pseudotime trajectory and RNA velocity analysis revealed that MSCs transformed into TSCs, which further differentiated into osteoblasts, hypertrophic chondrocytes and tenocytes. In addition, chondrocytes (CYTL1^high^ + THBS1^high^) from secondary cartilage were detected only in 4-month-old human embryonic TMJC.

**Conclusions:**

Our study provides an atlas of differentiation stages of human embryonic TMJC tissue cells, which will contribute to an in-depth understanding of the pathophysiology of the TMJC tissue repair process and ultimately help to solve clinical problems.

**Supplementary Information:**

The online version contains supplementary material available at 10.1186/s13578-023-01069-5.

## Background

During human embryonic development, abnormal development of the TMJC cartilage can lead to malformation of the oral and maxillofacial system, and abnormal respiratory and masticatory functions [1, 2]. Dysplasia development of the TMJ during the embryonic period may even lead to the development of congenital TMJ disorders. The TMJ is a complex joint that including the condyle, temporal articular surface, and the articular disc [1, 3]. Functions such as mastication, swallowing, and articulation are accomplished through TMJ movements [2, 4].

The embryonic development of human TMJ can be divided into three important stages: 1. the initial stage (8–9 weeks), the temporal and condylar embryonic bases appear, the condylar embryonic base begins intrachondral osteogenesis; 2. the differentiation stage (10–20 weeks), the articular disc, joint cavity and synovial membrane are formed, secondary cartilage appears, the condyle further endochondral osteogenesis; 3. The completion stage (21 weeks to delivery), the components of the TMJ have formed, Meckel's cartilage disappears, the articular disc is further modified, the condyle continues endochondral osteogenesis, endochondral osteogenesis occurs in the articular fossa, and haematopoietic tissue appears. Of these, the differentiation phase (10–20 weeks) is crucial for the development of the condylar tissue of the TMJ [2, 5]. A single-cell atlas of the condylar tissue of human embryos at three and four months of age was constructed. To our knowledge, this is the first and earliest single-cell atlas of human embryonic TMJ condylar tissue.

Single-cell RNA sequencing had been widely used in the study of joints. In the mouse knee joint, combining segmental bulk- and single-cell RNA sequencing were used to define the chondrocyte gene expression signature [6]. Single-cell RNA sequencing was used to comparing major cell clusters in osteoarthritic, Kashin-Beck disease and healthy articular chondrocytes [7]. Single-cell RNA sequencing has shown that different subpopulations of synovial fibroblasts exist in patients with rheumatoid arthritis (RA) [8]. Single-cell RNA sequencing revealed the transcriptomic changes in subchondral bone hypoplasia of the knee and in cartilage after traumatic fracture [9]. Single-cell RNA sequencing has also identified transcriptome heterogeneity and early molecular changes associated with post-traumatic osteoarthritis in mouse articular chondrocytes [10]. However, studies of single-cell RNA sequencing of the TMJ, particularly in human embryos, have not been reported.

Many key genes and transcription factors were reported to be involved in the differentiation of chondrocytes and osteoblasts. The common mesenchymal progenitors cell of chondrocytes and osteoblasts can express *Sox9* and *Runx2* [11]. The direction of cell differentiation toward chondrocytes or osteoblasts depends on the expression of *Sox9* and *Runx2* [12]. *Sox9* regulates the chondrocytes differentiation [13]. *Runx2* is expressed in hypertrophic chondrocytes and promotes the expression of *Col10a1* and *Mmp13* [14]. *Runx2* also promotes osteoblast differentiation by regulating the expression of *Osx* [15]. *Osx* transactivates *Col1a1*, which is essential for the differentiated osteoblasts. Therefore, *Osx* is essential for the differentiation of preosteoblastic cells [15]. Moreover, many signaling pathways have been reported to be involved in the differentiation of bone cells. The Indian Hedgehog (IHH) signaling pathway promotes chondrocyte differentiation toward hypertrophic terminal [16]. Conversely, the parathyroid hormone-related peptide (PTHrP) signaling pathway prevents premature chondrocyte differentiation [17]. In the previous studies, many other signaling pathways including PTN [18], BMP [19], FGF [20], CXCL [21], MSTN [22] and GH [23] have been associated with chondrocyte and osteoblast differentiation. In conclusion, the exploration of key genes, transcription factors and signaling pathways is important for understanding the development and differentiation of human embryonic TMJC cells.

In recent years, single-cell sequencing techniques have been used to study the cell types of tissues and the differentiation relationships between cell clusters. To study the cell types and differentiation relationships of human embryonic TMJC, we completed single-cell sequencing of TMJC from 3-and 4-month-old human embryos. To the best of our knowledge, this is the first and earliest single-cell atlas of human embryonic TMJC. In this study, we describe the cell types and differentiation relationships of human embryonic TMJC tissue. This has important implications for studying the functional and differentiation relationships of cell clusters and the congenital TMJ diseases caused by abnormal development of human embryonic TMJC.

## Results

### scRNA-Seq analysis of human embryonic TMJC cell types

**Fig. 1:**
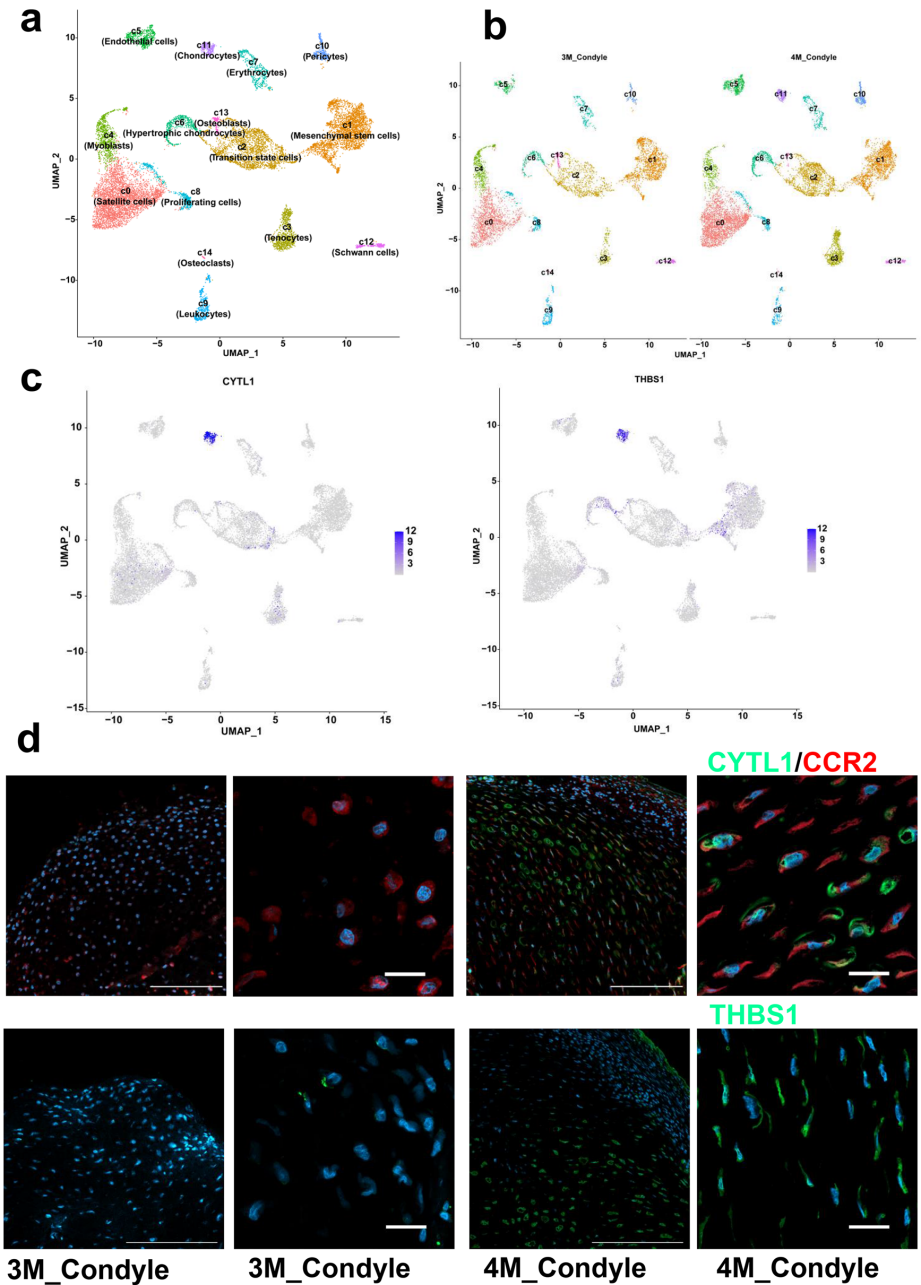
Overview of the scRNA sequencing of human embryonic TMJC. **a** The 15 cell clusters identified in human embryonic TMJC using UMAP. **b** The cell clusters were identified in 3 and 4-month-old human embryonic TMJC. **c** The expression of CYTL1 and THBS1 in each cluster of human embryonic TMJC. **d** The immunofluorescent staining of CYTL1 and THBS1 in 3 and 4-month-old human embryonic TMJC, Scale bar = 1 mm and 50 µm

To construct a cellular atlas of the human embryonic TMJC in 3 and 4-month-old, we isolated TMJC tissue from two early developmental stages: 7052 cells from 3-month-old TMJC and 9572 cells from 4-month-old TMJC. After cell quality control (Additional file 1: Fig. S1b), a total of 16,624 cells were clustered into 15 cells clusters. We annotated these 15 cell clusters to include satellite cells, MSCs, TSCs, tenocytes, myoblasts, endothelial cells, hypertrophic chondrocytes, erythrocytes, proliferating cells, leukocytes, pericytes, chondrocytes (CYTL1^high^ + THBS1^high^), schwann's cells, osteoblasts and osteoclasts (Fig. 1a and Additional file 1: Fig. S5). Unexpectedly, we found no chondrocytes cluster from secondary cartilage in the 3-month-old TMJC. However, a distinct cluster of chondrocytes (CYTL1^high^ + THBS1^high^) from secondary cartilage appeared in 4-month-old TMJC (Fig. 1b and Additional file 1: Fig. S1c). The marker genes used for annotation are shown (Additional file 1: Fig. S1d and Additional file 1: Fig. S6). The expression of CYTL1 and THBS1 as marker genes for chondrocytes clustering is illustrated (Fig. 1c). Immunofluorescence staining showed that CYTL1 and THBS1 were expressed in 4-month-old TMJC. CYTL1 and THBS1 were not detected in 3-month-old TMJC (Fig. 1d). The top 5 DEGs in each cluster are shown (Additional file 1: Fig. S1e). The GO analysis for each cluster is also shown (Additional file 1: Fig. S1f).

Marker genes used to annotate of hypertrophic chondrocytes included COL10A1, MMP13, RUNX2, FGFBP2 and SCIN (Additional file 2: Fig. S2a, 2b). Immunofluorescence staining showed FGFBP2 and SCIN were expressed in hypertrophic chondrocytes from 3-month-old and 4-month-old TMJC (Additional file 2: Fig. S2c).

### Subpopulation analysis of TSCs

**Fig. 2:**
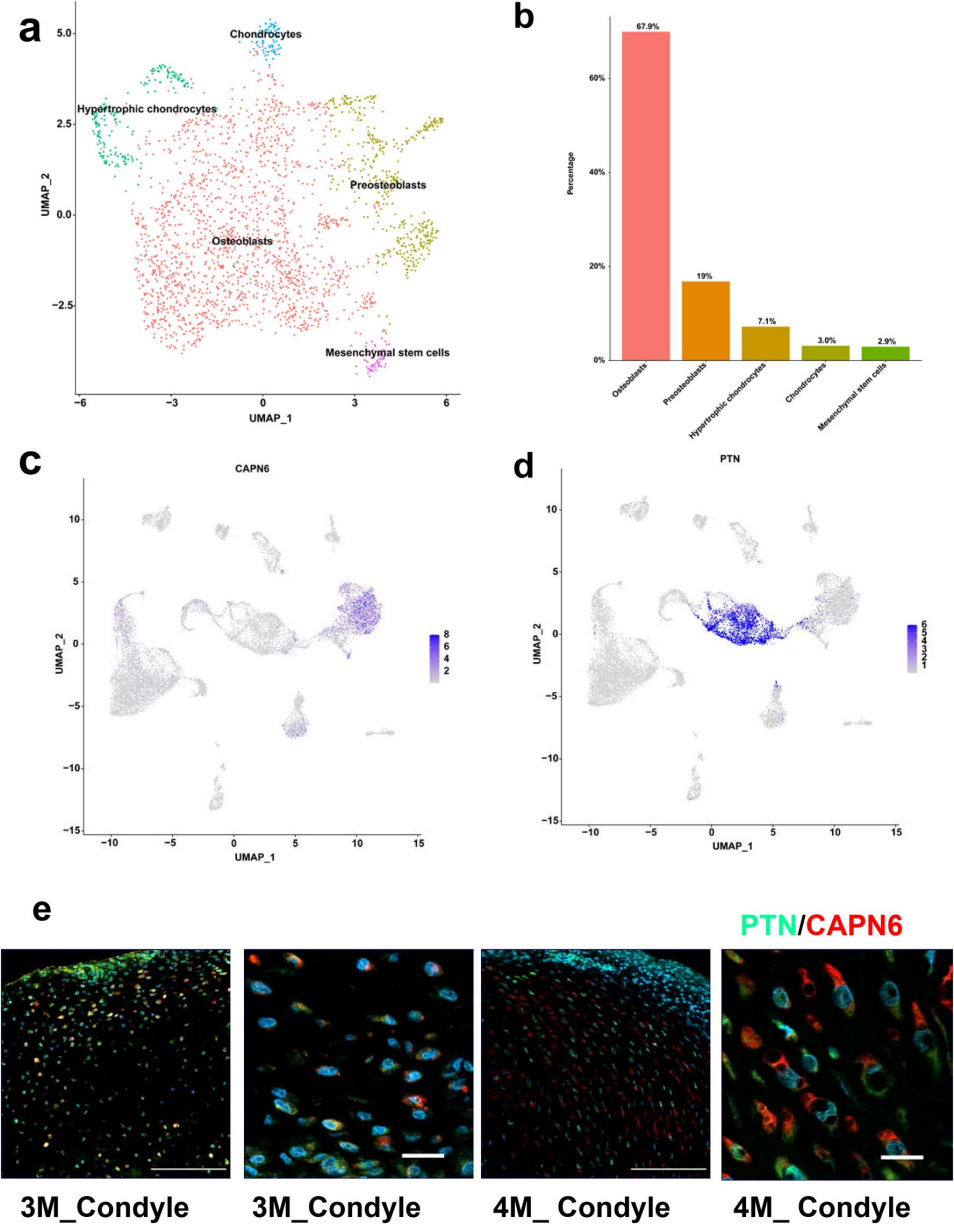
Subpopulation analysis of TSCs. **a** The five subpopulations of TSCs were visualized using UMAP. **b** The cell proportions of subpopulations in TSCs. **c** The expression of CAPN6 in each cluster of human embryonic TMJC. **d** The expression of PTN in each cluster of human embryonic TMJC. **e** Immunofluorescence staining of CAPN6 and PTN in 3 and 4-month-old human embryonic TMJC, Scale bar = 1 mm and 50 µm

In this study, we identified TSCs as a specific cell cluster. Subpopulation analysis revealed that TSCs consisted of 5 subpopulations (Fig. 2a). The proportions of the subpopulations were osteoblasts (67.9%), preosteoblasts (19.0%), hypertrophic chondrocytes (7.1%), chondrocytes (3.0%) and MSCs (2.9%) (Fig. 2b). Five subpopulations were annotated on the reported cell markers (Additional file 1: Fig. S1g). As marker genes, CAPN6 expression in MSCs and PTN expression in TSCs are illustrated separately (Fig. 2c, d). Immunofluorescence staining showed no co-localization and expression of CAPN6 and PTN in different cells of 3 and 4-month-old TMJC (Fig. 2e). These results indicate the presence of MSCs and TSCs in 3 and 4-month-old TMJCs. These results also further confirm that MSCs and TSCs are distinct cell types in human embryonic TMJC (Fig. 2e).

### Monocle3 and RNA velocity analysis of cell differentiation relationships

**Fig. 3:**
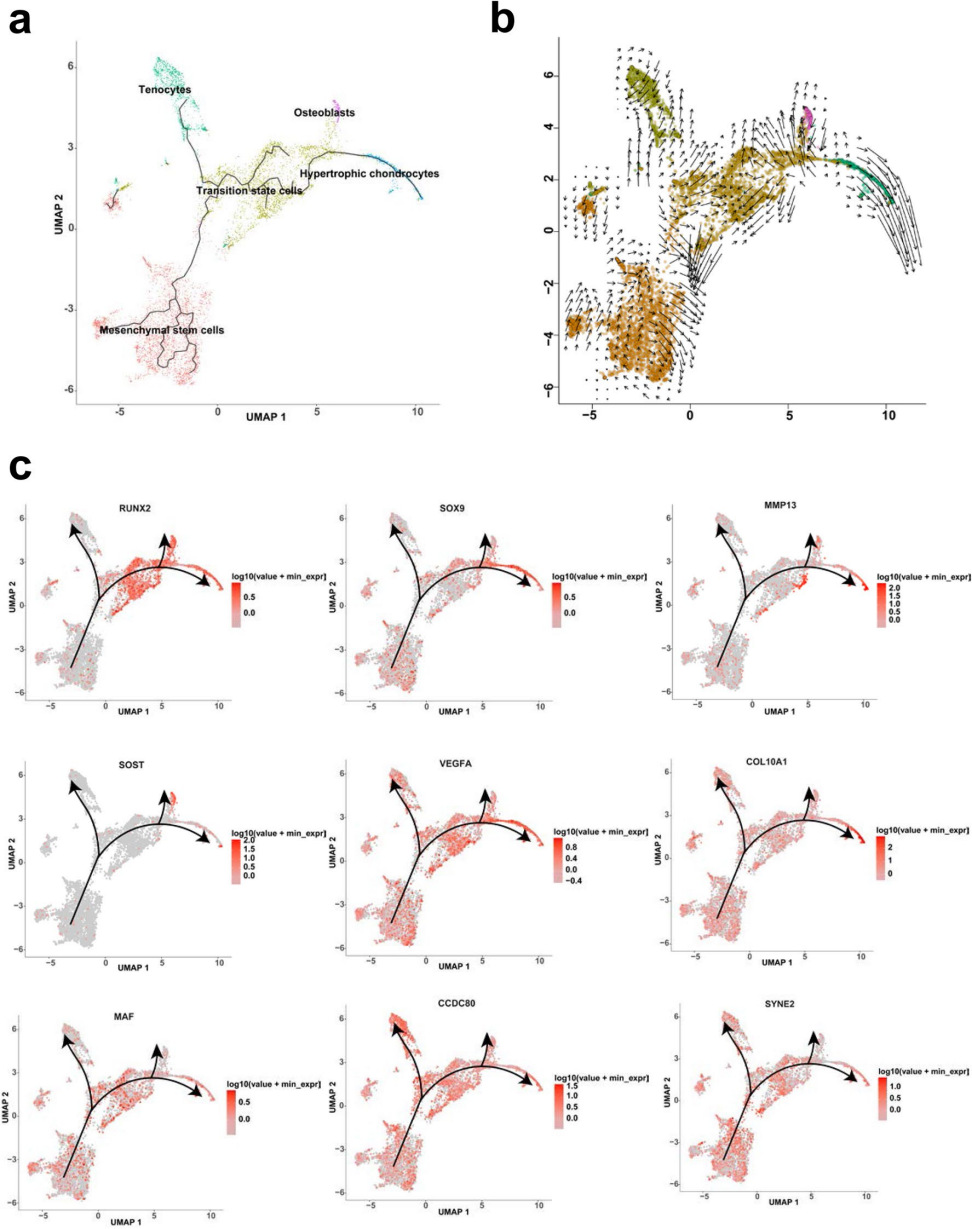
The differentiation relationship analysis of MSCs, TSCs, tenocytes, hypertrophic chondrocytes and osteoblasts. **a** The differentiation relationship among MSCs, TSCs, tenocytes, hypertrophic chondrocytes and osteoblasts based on Monocle3 analysis. **b** The differentiation relationship among these five clusters based on RNA velocity analysis. **c** The expression of key genes including RUNX2, SOX9, MMP13, SOST, VEGFA, COL10A1, MAF, CCDC80 and SYNE2 among the five clusters

In order to clarify the differentiation relationships between TMJC cells, 15 TMJC cell clusters were calculated and divided into 8 differentiation trajectories based on Monocle3 analysis. Clear differentiation pathways could be found between MSCs, TSCs, tenocytes, hypertrophic chondrocytes and osteoblasts (Additional file 3: Fig. S3a). We further focused on the differentiation relationships between these five cell clusters. It is reasonable to designate the MSC cluster as the root in the pseudotime trajectory pathway. The differentiation relationships between these five cell clusters were identified. MSCs were positioned as the origin and differentiated into TSCs. TSCs could further differentiate into tenocytes, hypertrophic chondrocytes and osteoblasts, respectively (Fig. 3a). Pseudotime analysis of 3- and 4-month-old TMJC cells is also shown (Additional file 3: Fig. S3c). In addition, we used RNA velocity analysis to look at the extent and orientation of MSCs, TSCs, tenocytes, hypertrophic chondrocytes and osteoblasts. The arrow directions showed that MSCs and TSCs are highly heterogeneous and are thought to be the starting point for differentiation in each direction. The arrowheads of the tenocytes, hypertrophic chondrocytes and osteoblasts are oriented in the same direction, indicating that these three clusters have a stable state and appear to differentiate from the TSCs (Fig. 3b). RNA velocity analysis of 15 TMJC cell clusters and 5 cell clusters from 3- and 4-month-old TMJCs are also shown (Additional file 3: Fig. S3b, d), and these results are consistent with the monocle3 analysis. Expression of key genes associated with chondrocyte and osteoblast differentiation were illustrated. These genes included RUNX2, SOX9, MMP13, SOST, VEGFA, COL10A1, MAF, CCDC80, and SYNE2 (Fig. 3c).

### CellChat analysis of signaling pathways in cell cluster differentiation

**Fig. 4:**
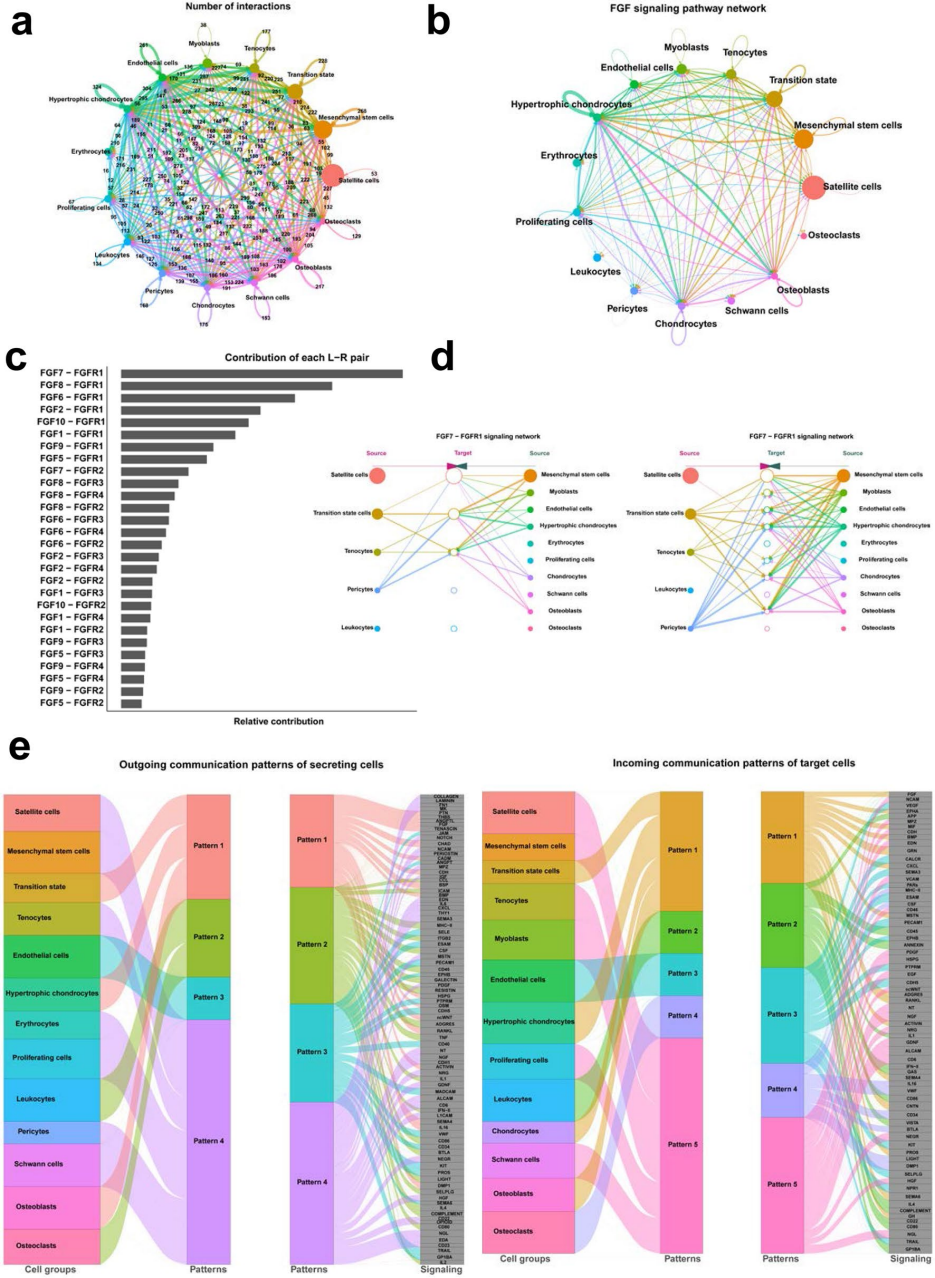
CellChat analysis of human embryonic TMJC clusters. **a** The number of ligand-receptor pairs in 15 cell clusters. **b** FGF signaling pathway networks among 15 cell clusters. **c** The contribution of each ligand-receptor pairs of FGF signaling pathway. **d** FGF7-FGFR1 signaling pathway networks among 15 cell clusters. **e** The analysis of outgoing communication patterns of secreting cells and incoming communication patterns of target cells among 15 cell clusters

To analyze cell–cell interactions and potential signaling pathways, we screened known cell–cell exchanges using CellChat. Complex networks of cell–cell interactions were identified in 15 cell clusters (Fig. 4a and Additional file 4: Fig. S4a). Notably, the FGF signaling pathway inferred by CellChat was highly enriched in MSCs, TSCs, hypertrophic chondrocytes osteoblasts and tenocytes (Fig. 4b and Additional file 4: Fig. S4b). We further identified FGF7-FGFR1 as a major contributor to the FGF signaling pathway (Fig. 4c). We further investigated the expression of molecules involved in FGF7-FGFR1 and found that FGFR1 was mainly expressed in TSCs, tenocytes, hypertrophic chondrocytes and osteoblasts (Additional file 4: Fig. S4c). Thus, FGF7-FGFR1 is the main signaling pathway mediating cell–cell communication between MSCs, TSCs, hypertrophic chondrocytes, osteoblasts and tenocytes (Fig. 4d and Additional file 4: Fig. S4d). In addition, we also demonstrated the other key signaling pathways with the function of “identify Communication Patterns” in CellChat. Among the patterns of outgoing communication in secretory cells, TSCs, hypertrophic chondrocytes and osteoblasts belong to pattern 1. Pattern 1 contains PTN, THBS, ANGPTL, FGF, TENASCIN, CHAD, CADM, CDH, BSP, BMP, HSPG, OSM, ncWNT, RANKL, ACTIVIN, SEMA4, IL16, and DMP1 and other signaling pathways. Meanwhile, incoming communication patterns of target cells indicated that TSCs, hypertrophic chondrocytes and osteoblasts were in pattern 1, regulated by FGF, EPHA, MIF, BMP, GRN, CXCL, MSTN, ADGRE5, RANKL, ACTIVIN, SEMA4, CNTN, PROS, SEMA6 and GH (Fig. 4e and Additional file 4: Fig. S4e).

### Analysis of transcription factors in cells cluster differentiation

**Fig. 5:**
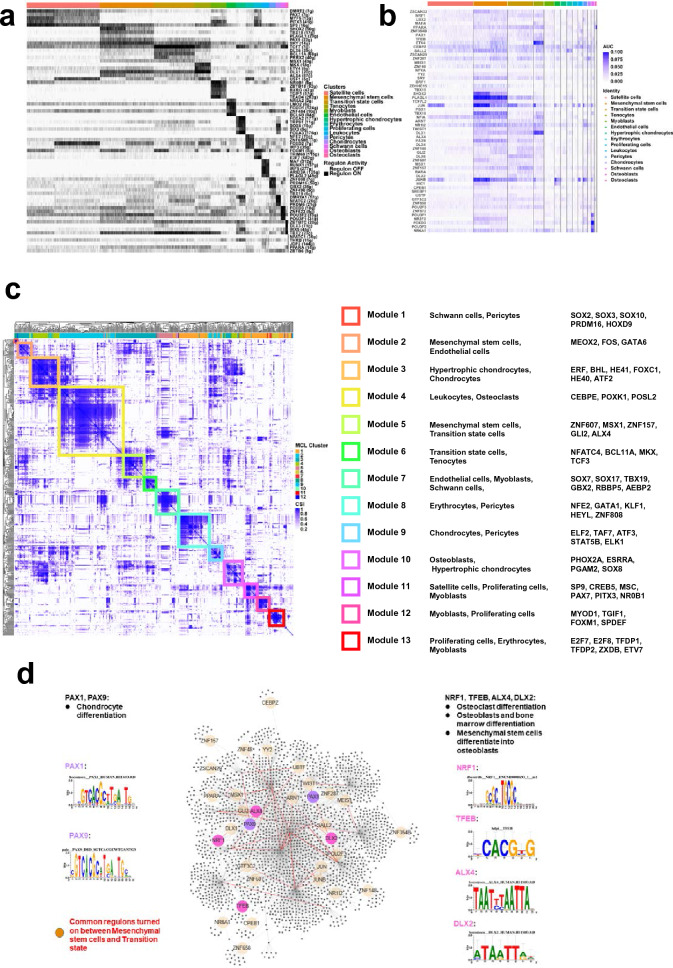
Transcription factors analysis among human embryonic TMJC clusters. **a** The regulon activity analysis showed the on/off of the specific regulons in each cluster. **b** Heatmap showed the common regulons were turned on between MSCs and TSCs. **c** Modules analysis of cell clusters and common regulons. **d** Interaction mapping of transcription factors networks showed common regulons between MSCs and TSCs

Figure 5a shows the activity matrix of binary regulators. SP9, SHOX2, TBX18, PLAGL1, PAX9 and SRY are turned on in MSCs. TCF7, DLX6, BCL11A, PRRX2 and MSX1 are turned on in TSCs. MKX, ETV4, DLX1, ALX4 and USF1 are turned on in tenocytes. FOXA2, TRPS1, SOX6, SIX3 and FOXA3 are turned on in hypertrophic chondrocytes. ZBTB7C, DLX3, IRX5 and TBX2 are turned on in osteoblasts. Strikingly, all 483 regulons were organized into 13 modules. Representative regulators and cell clusters were identified in each module. For example, module 3 consisted of hypertrophic chondrocytes and chondrocytes, containing regulons of ERF, BHL, HE41, FOXC1, HE40 and ATF2. Module 5 was organized by MSCs and TSCs and included ZNF607, MSX1, ZNF157, GLI2 and ALX4. Module 10 consisted of hypertrophic chondrocytes and osteoblasts, containing regulators of PHOX2A, ESRRA, PGAM2 and SOX8 (Fig. 5c). The heatmap shows that many common regulators are turned on between MSCs and TSCs (Fig. 5b). Interaction mapping of transcription factor networks showed that PAX1 and PAX9 are important regulators of chondrocyte differentiation and are commonly switched on between MSCs and TSCs. NRF1, TFEB, ALX4 and DLX2 are associated with the differentiation of MSCs into osteoblasts and also commonly switched on between MSCs and TSCs. In addition, sequences of promoter regions of transcription factors were shown to target genes, including PAX1, PAX9, NRF1, TFEB, ALX4 and DLX2 (Fig. 5d).

## Discussion

During the embryonic period, the development of the TMJ lags behind that of other joints [24]. As a result, at birth, the TMJ remains largely immature. Developmental disorders of the TMJ such as hypoplasia and aplasia are characterized by TMJ dysfunction [25]. In addition, there are many causes of TMJ growth disorders and abnormalities. Growth disorders of TMJ development may occur in utero and lead to contributions such as hyperplasia or hypoplasia of the TMJ. Congenital abnormalities of the TMJ can be divided into three categories, including hypoplasia or aplasia of the TMJ, hyperplasia and bifidity [24, 26]. It is important to study the development of the condyle for treating congenital abnormalities of the condyle.

Single-cell RNA sequencing was used to identify new cell clusters and cell function in the joint. Seven clusters of articular chondrocytes in human OA cartilage have been identified by single-cell RNA sequencing [27]. Different subtypes of cells, particularly macrophages, have been shown to have pro- or anti-inflammatory effects in the joint depending on polarization [28, 29]. In patients with rheumatoid arthritis (RA), 13 distinct cell subpopulations were identified within the synovium by single-cell RNA sequencing [30]. In the adult knee synovium, the Gdf5-lineage cell cluster contains fibroblasts that become pathogenic in inflammatory arthritis [31]. In the articular cartilage of the knee joint of healthy mice, nine chondrocyte subtypes were identified by single-cell RNA sequencing [10]. Single-cell RNA sequencing was used to determine the differentiation trajectory and molecular regulation of fibroblast and progenitor cell clusters in the synovial membrane of knee joints of steady-state and injured mouse knees [32]. In addition to the common cell populations of Osteoblast, Chondrocyte, Tenocyte, there are cell population difference between the synovial membrane of the knee joints and TMJC.

To better understand the cell types and differentiation relationships of the human embryonic TMJC, we constructed a cellular atlas of the 3- and 4-month-old TMJC by single-cell sequencing. In previous reports, many studies have revealed the development of TMJ through imaging and morphological studies [5]. To date, there have been no studies on the cellular level of the human embryonic TMJC. This is the first and earliest atlas of the cellular development of the human embryonic TMJC. We have identified 15 cell clusters (Fig. 1a). We found that the chondrocytes cluster (CYTL1^high^ + THBS1^high^) was not yet differentiated in 3-month-old TMJC. However, a distinct chondrocytes population emerged in 4-month-old TMJC (Fig. 1b and Additional file 1: Fig. S1c). We therefore, hypothesize that chondrocytes have differentiated in 4-month-old TMJC. Chondrocytes differentiate later than other cell clusters, suggesting that chondrocytes differentiation is completed at a later stage of TMJC development. This phenomenon is the first to be reported in the development of human embryonic TMJC.

We identified TSCs as a new cell cluster that can be divided into five subpopulations including osteoblasts, preosteoblasts, hypertrophic chondrocytes, chondrocytes and MSCs (Fig. 2a). The different composition of TSCs suggests that TSCs possess multiple differentiation potentials. Based on this prediction, three new differentiation lineages related to TSCs were proposed by trajectory and RNA velocity analysis. MSCs differentiate into TSCs. TSCs can further differentiate into hypertrophic chondrocytes, osteoblasts and tenocytes (Fig. 3a, b). MSCs have been reported to differentiate into tenocytes [33]. Contrast to previous reports [33], tenocytes were not differentiated directly from MSCs, but from TSCs from human embryonic TMJC (Fig. 3a). The stature-deficient homeobox 2 (*Shox2*) has been reported to be expressed in the developing TMJ. Overexpression of *Shox2* will result in congenital hypoplasia in the TMJ in mice. Furthermore, deletion of *Shox2* also leads to hypoplasia in the TMJ of mice. In our study, SHOX2 was specially expressed in MSCs of human embryonic TMJC. These results suggest that SHOX2 plays an important role in the function and early differentiation of the MSCs in human embryonic TMJC. During development, hypertrophic chondrocytes and osteoblasts have been reported as separate cell lines [11]. They are differentiated from osteochondro-progenitors cells. *Sox9* and *Runx2* are highly expressed in osteochondro-progenitors cells [34]. In the present study, similar to osteochondro-progenitors, TSCs highly expressed *Sox9* and *Runx2* and were able to differentiate into hypertrophic chondrocytes and osteoblasts, respectively (Fig. 3c). In addition, RUNX2 [35] has been reported to be involved in the trans-differentiation of chondrocytes to osteoblasts and to promoted osteoblast differentiation. RUNX2 is highly expressed in the differentiation pathways from TSCs to hypertrophic chondrocytes and from TSCs to osteoblasts. SOX9 has been reported to be a key transcriptional regulator of chondrogenesis [36]. MMP13 has been implicated in osteogenesis [37]. SOX9 and MMP13 are highly expressed in the differentiation of TSCs to hypertrophic chondrocytes. SOST has been implicated in the differentiation of bone formation [38]. In the present study, SOST was highly expressed in osteoclasts. Other key genes include VEGFA [39], COL10A1 [40], MAF [41], CCDC80 [42] and SYNE2 [43] which are associated with bone proliferation and differentiation and are widely expressed in MSCs, TSCs, tenocytes, hypertrophic chondrocytes and osteoblasts. These results suggest the key genes associated with the differentiation of chondrocytes and osteoblasts are expressed in the TMJC clusters (Fig. 3c). These genes can promote the differentiation of human embryonic TMJC cells. Interestingly, we found that TSCs also have the potential ability to differentiate into tenocytes. Thus, as intermediate cells, between MSCs and other cell clusters, TSCs are critical for the development of human embryonic TMJCs. Although we have established the presence of TSCs in the human embryonic TMJC (Fig. 2e), further experiments are required to reveal their detailed functions.

We performed an analysis of cell–cell communication between the TMJC cell clusters. We found that the FGF signaling pathway mediated the communication between these five clusters (Fig. 4b). We then analyzed the strength of the FGF signaling pathway and found that FGF7-FGFR1 was the main pathway (Fig. 4c, d). FGF7 has been reported to promote bone formation by increasing osteogenesis [44]. FGF7 also promotes osteogenic differentiation by regulating the expression of the β-catenin and Runx2 signaling pathways [45]. In the present study, as a source cell cluster, TSCs communicated with target cell cluster of MSCs via the FGF7-FGFR1 signaling pathway. In addition, as source cell clusters, hypertrophic chondrocytes, osteoblasts and tenocytes also communicate with the target cell clusters of TSCs via the FGF7-FGFR1 signaling pathway, respectively. Thus, as the primary signaling pathway, we determined that FGF7-FGFR1 mediated the communication and differentiation among MSCs, TSCs, hypertrophic chondrocytes, osteoblasts and tenocytes. In addition, we explored other signaling pathways between TSCs, hypertrophic chondrocytes and osteoblasts. Notably, we screened many chondrocyte and osteoblast differentiation signaling pathways, including PTN [18], BMP [19], TENASCIN [46], ACTIVIN [47], RANKL [47], DMP1 [48], MK [49], FGF [20], GRN [50], CXCL [21], MSTN [22] and GH [23]. These reported signaling pathways regulating bone differentiation may also play an important role in the differentiation of TSCs, hypertrophic chondrocytes and osteoblasts. In addition, we screened other signaling pathways including THBS, ANGPTL, CHAD, CADM, CDH, BSP, HSPG, OSM, ncWNT, SEMA4, IL16, which also mediate cellular communication in TSCs, hypertrophic chondrocytes and osteoblasts (Fig. 4e). The role of these signaling pathways in cell differentiation is elusive and needs to be further explored.

In addition to cell–cell communication analysis, transcription factor analysis revealed that some specific transcription factors were turned on in the cell clusters (Fig. 5a). For example, SHOX2 [51], TBX18 [52] and PAX9 [53], which are associated with ossification and chondroitin differentiation, are turned on in MSCs. TCF7 [20], DLX6 [54] and MSX1 [55], which are reported to be involved in ossification and chondroitin differentiation, are turned on in TSCs. MKX [56] which is associated with tenocytes differentiation, is turned on in tenocytes. FOXA2 [57], TRPS1 [58], SOX6 [59] and FOXA3 [60], which are associated with chondrocyte differentiation and hypertrophy, are turned on in hypertrophic chondrocytes. DLX3 [61] and IRX5 [62] which are associated with the ossification and differentiation, are turned on in osteoblasts. These results suggest that transcription factors that are specifically switched on are essential for maintaining specific functions of the cell cluster. Furthermore, module analysis revealed that common regulatory factors were switched on in different cell clusters (Fig. 5c). For example, Module 3 consists of hypertrophic chondrocytes and chondrocytes containing common regulators of ERF [63], FOXC1 [64] and ATF2 [65] which have been reported to be involved in bone formation and osteogenic differentiation. Module 10 consists of hypertrophic chondrocytes and osteoblasts and contains co-regulators of SOX8 [66] and ESRRA [67] which are important regulators of chondrogenic and osteoblast differentiation (Fig. 5c). Notably, co-regulators including PAX9 [53], SP9 and TCF7 [20, 68] were switched on between MSCs and TSCs. Regulon module analysis showed MSCs and TSCs were in module 5. 32 co-regulators were further switched on between MSCs and TSCs. Among these co-regulators, PAX1 [69] and PAX9 are important regulators of chondrocyte differentiation. NRF1 [70], TFEB [71], ALX4 [72] and DLX2 [73] are associated with the differentiation of MSCs into osteoblasts (Fig. 5d). These co-regulators turned on between MSCs and TSCs may mediate the differentiation from MSCs to TSCs and further to tenocytes, hypertrophic chondrocytes, and osteoblasts, respectively.

## Conclusions

**Fig. 6:**
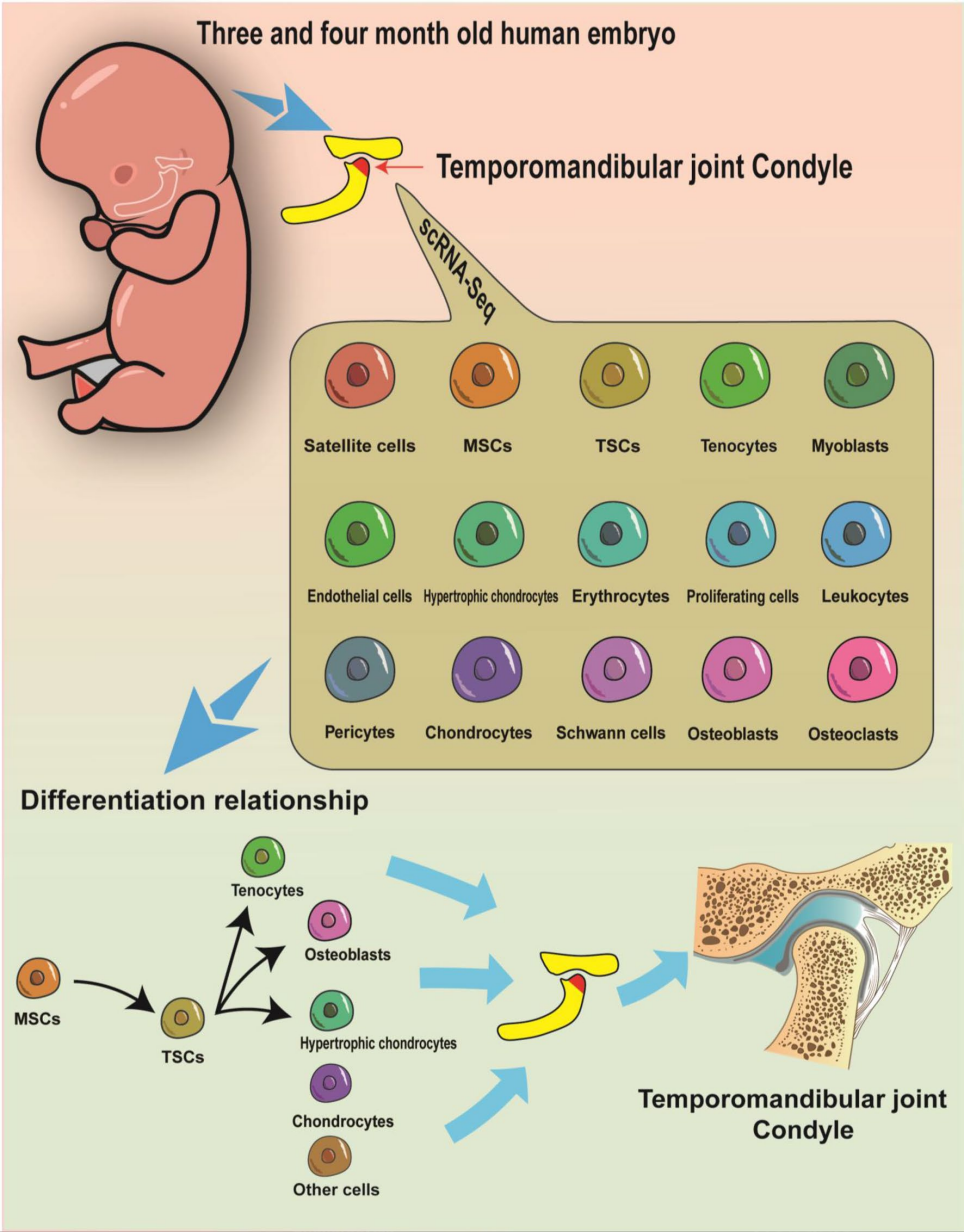
Overview of the cell types and differentiation relationship among human embryonic TMJC cells

In summary, our data provided the first and earliest cellular atlas of the human embryonic TMJC. The present results show that chondrocytes from secondary cartilage differentiate in 4-month-old TMJC and that chondrocytes differentiate later than other cell clusters. Our results also demonstrated the differentiation relationships and underlying mechanisms of differentiation between MSCs, TSCs, hypertrophic chondrocytes, osteoblasts and tenocytes by advanced analysis of single-cell sequencing (Fig. 6). This study is an indispensable resource for studying the development and differentiation of human embryonic TMJC. Our findings also have important implications for the study of cellular mechanisms of temporomandibular joint disorders (TMD), which are caused by abnormal embryonic development of the TMJC.

## Methods

### Collection of human embryonic TMJC

This work was reviewed and approved by the Institutional Review Boards of Nantong University Hospital (2020-K013). Parents of study participants signed an Informed consent form. For scRNA-seq, TMJC tissue was isolated by surgical excision. The entire mandible, including the mandibular body and TMJ (condyle, joint capsule, joint disc, part of fibrous ligament and temporo-articular fossa), was isolated along the mandibular labiobuccal migration. The mandibular body, condyle and coracoid process were separated from the rest of the TMJ. The condyle is separated from the mandibular body through the neck of the condyle (Additional file 1: Fig. S1a). The left condyle was cut into pieces as far as possible and placed in tissue preservation solution (Singleron Biotechnologies, Nanjing, China), and then transported to the laboratory by cold chain for cell dissociation and single cell sequencing.

### Preparation of single-cell suspensions

The dissociation of human embryonic TMJC was performed according to a previous study [74]. Briefly, the isolated TMJC tissue was cut into 2–3 mm pieces. Tissue pieces were washed with Hanks Balanced Salt Solution (HBSS). These tissue fragments were spin-digested for 15 using tissue dissociation solution (Singleron Biotechnologies, Nanjing, China). The pieces were filtered through a sterile 40-μm filter (Corning, NY, USA) and centrifuged at 150 g for 5 min. Erythrocytes were removed using a lysis reagent (Singleron Biotechnologies, Nanjing, China) for 10 min. Afterwards, cells were resuspended in PBS and stained with trypan blue (T6146, Sigma, Burlington, VT, USA). Cell viability was assessed using a TC20 automated cell counter (Bio-Rad, Hercules, CA, USA).

### Library preparation and data pre-processing

A cell suspension at a concentration of 1 × 10^5^ cells/mL of was then added to the microfluidic plate. The scRNA-seq libraries were constructed using the Single-cell RNA Library Kit (Singleron Biotechnologies, Nanjing, China). The constructed libraries were pooled onto an Illumina HiSeq × 10 sequencer machine for sequencing. Raw reads were processed by the CeleScope (https://github.com/singleron-RD/CeleScope) pipeline. First, low quality raw reads and adaptor sequences were trimmed by fastqc (version 0.11.7) and cutadapt (version 1.17). Reads were mapped to GRCm38 (Ensembl V. 92 annotation) using STAR (version 020201). Gene counts and UMI counts were calculated using FeatureCounts (version 1.6.2). A series of analysis procedures were completed by CeleScope and a gene expression matrix was generated.

### Dimension-reduction and clustering analysis

The dimension reduction and cell clustering were performed in R using the Seurat version 4 package (https://satijalab.org/seurat/) [75]. Sctransform (SCT) (https://github.com/ChristophH/sctransform) was used to remove batch effects and to normalize the expression data. The function of subset was used to extract a subset of in Seurat objects which meet the requirements of quality control (nFeature_RNA > 200 & nFeature_RNA < 4000 & percent.mt < 50 & nCount_RNA < 20,000). The low-quality cells were filtered out and did not participate in the downstream data analysis. The first 3000 variable genes were selected for sample data integration. Cell clusters were separated suing “FindNeighbors” in 50 dimensions and “FindClusters” at a resolution of 0.25. Subcluster analyses of TSCs was set up using a resolution of 0.1. The clusters were visualized using the Uniform Manifold Approximation and Projection (UMAP) algorithm. For the analysis of differentially expressed genes (DEGs), Seurat’s “FindMarkers” was used. Genes that were more than 25% expressed in their clusters and had a mean log (Fold Change) greater than 0.25 were defined as DEGs.

### Cell type annotation and gene enrichment analyses

Cell types were annotated based on the expression of marker genes known from published literature. Subsequently, gene enrichment was performed using clusterProfiler v3.6.1 (https://bioconductor.org/packages/3.11/bioc/html/clusterProfiler.html) [76]. Biological process (BP) with p_adj value < 0.05 were defined as significantly enriched. GO classification was screened from the org.Hs.eg.db database (https://bioconductor.org/packages/3.11/data/annotation/html/org.Hs.eg.db.html).

### Trajectory analysis and RNA velocity

The pseudotime trajectories of TMJC cell clusters were analyzed by Monocle 3 (https://cole-trapnelllab.github.io/monocle3). Highly variable genes were selected from each cluster. Trajectories were visualized using the UMAP method. Finally, cells were sorted according to developmental and differentiation relationships. For RNA velocity analyses, the BAM file containing each cluster was first processed into a loom file. Subsequently, the loom files were processed as input into spliced and unspliced matrices. Finally, the results were visualized using the UMAP method.

### Analysis of cell–cell communication

Cell–cell communication networks for ligand-receptor interaction were analyzed using CellChat (http://www.cellchat.org/; last accessed on May 10, 2021) [77]. A total of 32,491 ligand-receptor pairs were screened from the “CellChatDB.human” database. Using the “computeCommunProb” and “aggregateNet” in CellChat, the number of ligand-receptor interactions and the strength of interactions in the TMJC cell population were calculated. The “computeCommunProbPathway” function was used to determine the major signaling pathways. The outgoing communication patterns of secretory cells and the incoming communication patterns of target cells were analyzed using “identifyCommunicationPatterns” function.

### Transcription factors analysis

Transcription factors were analyzed using a python version of Single-Cell Regulatory Network Inference and Clustering (pySCENIC) [78]. Cell-type-specific regulons were screened using the regulon specificity score (RSS) proposed in a previous study [79]. Regulon module analyses were performed using the Connection Specificity Index (CSI) parameter which has been reported in previous studies [80]. Regulatory networks associated with MSCs and TSCs were analyzed using Cytoscape [81].

### Immunofluorescent staining

The procedure for immunofluorescence was based on a previous studies [74]. Briefly, human embryonic TMJC was fixed in 4% paraformaldehyde for 24 h at 4 ℃. The samples were demineralized at 4 ℃ gently shaking for 2 weeks in 4% EDTA in PBS.A 4-μm-thick tissue section was cut from TMJC tissue. Antigen retrieval was performed using 10 mM sodium citrate buffer (pH 6.0). Sections were then blocked with 5% donkey serum containing 0.1% Triton X-100 in 2% BSA. Diluted primary antibodies were added to the sections and stored overnight at 4 ℃. Sections were further incubated in the dark with the secondary antibody for 1 h at room temperature. Cell nuclei were stained with DAPI for 5 min. The images were captured with Leica SP8 laser scanning confocal microscopy (Leica TCSSP8, Leica).

The primary antibodies including Calpain 6 (10,120-1-AP, Proteintech), FGFBP2 (13,254-1-AP, Proteintech), SCIN (11,579-1-AP, Proteintech), CCR2 (16,153–1-AP, Proteintech), CYTL1 (15,856-1-AP, Proteintech), Calpain 6 (MA5-24,733, Invitrogen), PTN (27,117-1-AP, Proteintech), PTN (H00005764-M01, Novus Biologicals), THBS1 (PA5-102,583, Invitrogen), THBS1 (18,304-1-AP, Proteintech), were used in this study. The second antibodies including Alexa Fluor^™^ 488 (A-21206, Invitrogen) and Alexa Fluor^™^ 568 (A10037, Invitrogen) were used in this study.

## Supplementary Information

**Additional file 1: Fig. S1. **Quality control of scRNA-seq and GO enrichment analysis.** a** The separation of TMJC. **b** The analysis of the number of genes, counts and percentage of mitochondria. **c** The statistics of cells in each cluster from 3 and 4-month-old human embryonic TMJC. **d** The markers genes for the annotation which were from pieces of literature. **e** The top five DEGs of each cluster in human embryonic TMJC. **f** GO analysis of each cluster in human embryonic TMJC. **g** The marker genes were used for the annotation of TSCs.

**Additional file 2: Fig. S2. **The immunofluorescent staining in 3 and 4-month-old human embryonic TMJC.** a** The expression of COL10A1, MMP13 and RUNX2 in each cluster of human embryonic TMJC. **b** The expression of FGFBP2 and SCIN in each cluster of human embryonic TMJC. **c** Immunofluorescence staining of FGFBP2 and SCIN in 3 and 4-month-old human embryonic TMJC, Scale bar = 1 mm and 50 µm.

**Additional file 3: Fig. S3. **The differentiation relationship among human embryonic TMJC cells. **a** The differentiation relationship among 15 TMJC cell clusters based on Monocle3 analysis. **b** The RNA velocity analysis of 15 TMJC cell clusters. **c** The pseudotime analysis among MSCs, TSCs, tenocytes, hypertrophic chondrocytes and osteoblasts of the 3 and 4-month-old human embryonic TMJC. **d** The RNA velocity analysis among MSCs, TSCs, tenocytes, hypertrophic chondrocytes and osteoblasts of the 3 and 4-month-old human embryonic TMJC.

**Additional file 4:** **Fig. S4. **Cell Chat analysis of human embryonic TMJC cell clusters.** a** The interaction strength analysis of ligand-receptor pairs in 15 cell clusters. **b** FGF signaling pathway networks among 15 cell clusters. **c** The expression analysis of FGFR1 among 15 cell clusters. **d** FGF7-FGFR1 signaling pathway networks among 15 cell clusters. **e** The analysis of outgoing signaling patterns and incoming signaling patterns among 15 cell clusters.

**Additional file 5. **

**Additional file 6. **

## Data Availability

The raw data for this study are deposited in the Genome Sequence Archive [82] at the National Genomics Data Center for Biological Information/National Genomics Data Center [83], Institute of Genomic Research, Chinese Academy of Sciences, Beijing, China (GSA-Human: HRA002545). The cell–gene matrix has been uploaded to “FigShare [https://doi.org/10.6084/ m9. Figshare.20407371, https://doi.org/10.6084/m9.figshare.20407383]. All other relevant data supporting the key findings of this study are available within the article and its Supplementary Information file, or from the corresponding author upon reasonable request.

## Code availability

All code associated with this manuscript have been uploaded to “GitHub [https://github.com/qianqi234/human_embryonic_TMJ]”.
