# Supplementary figures and images for: Single-cell RNA sequencing analysis of the temporomandibular joint condyle in 3 and 4-month-old human embryos
Source: Cell Biosci. 2023 Jul 19;13:130. doi: 10.1186/s13578-023-01069-5 (PMC10357633; doi:10.1186/s13578-023-01069-5)

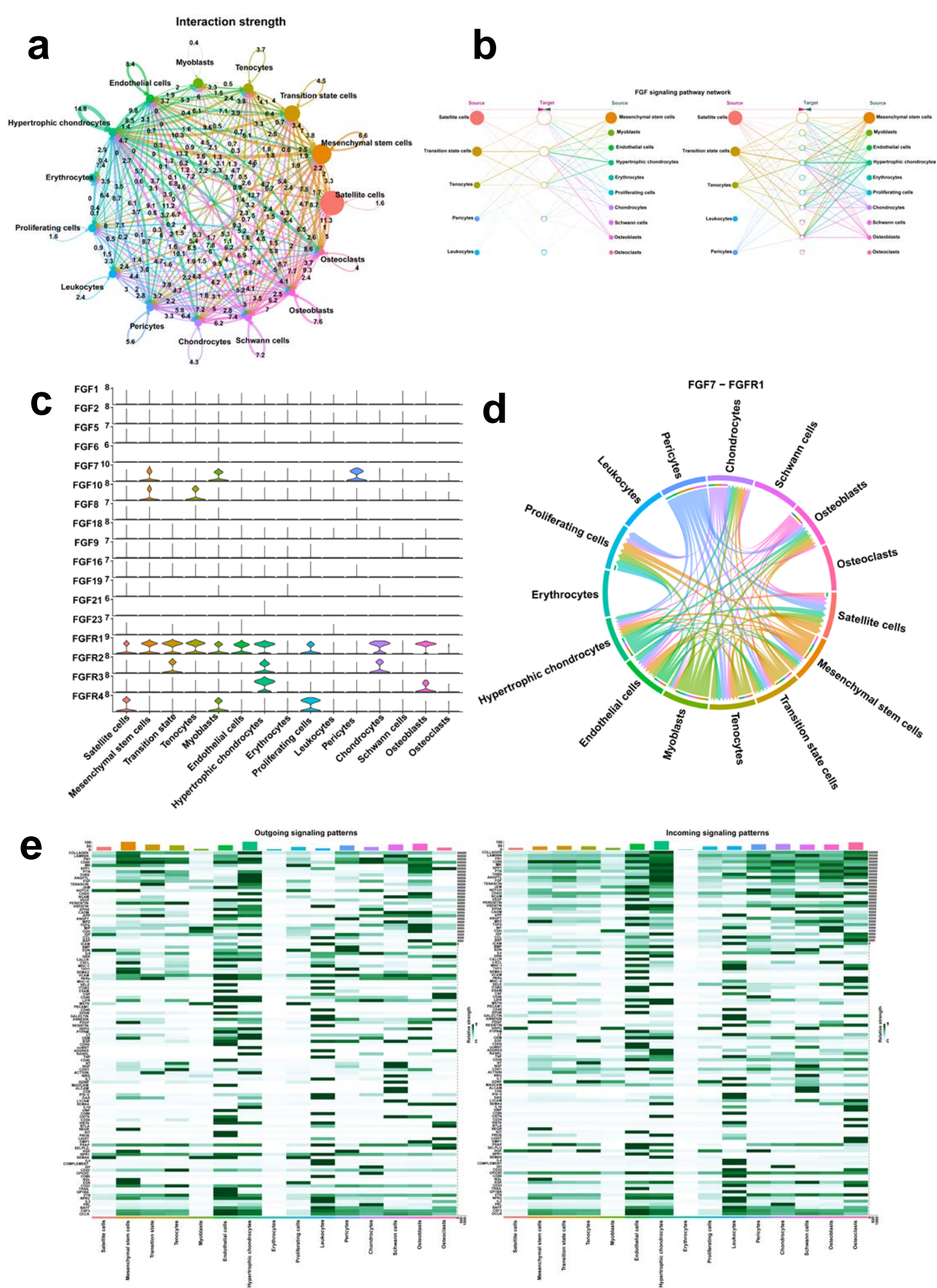

**Fig. S4**

Supplement: Supplementary file 1 — Additional file 1: Fig. S1. Quality control of scRNA-seq and GO enrichment analysis. a The separation of TMJC. b The analysis of the number of genes, counts and percentage of mitochondria. c The statistics of cells in each cluster from 3 and 4-month-old human embryonic TMJC. d The markers genes for the annotation which were from pieces of literature. e The top five DEGs of each cluster in human embryonic TMJC. f GO analysis of each cluster in human embryonic TMJC. g The marker genes were used for the annotation of TSCs. [file 13578_2023_1069_MOESM1_ESM.pdf]

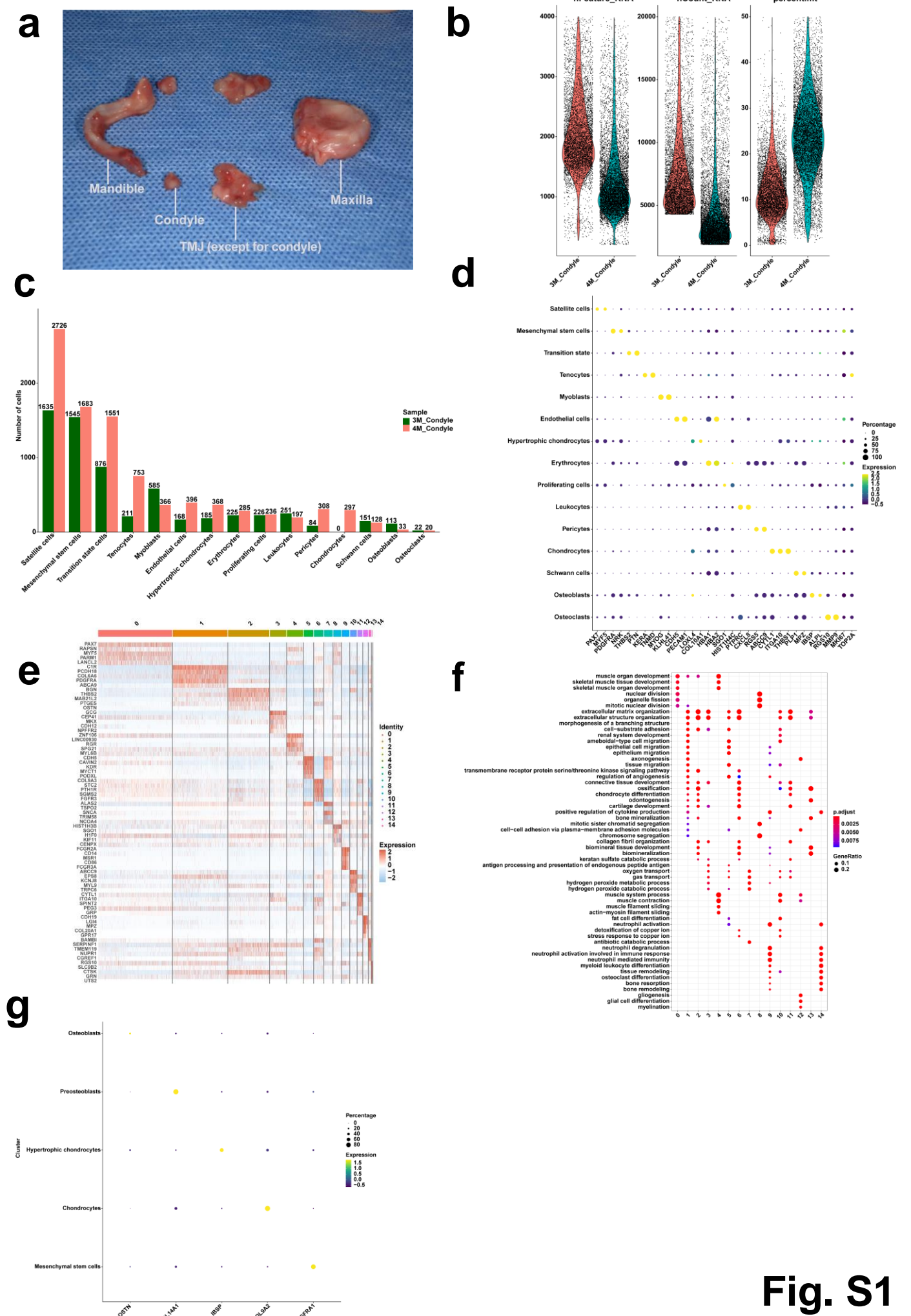

Fig. S1

Supplement: Supplementary file 2 — Additional file 2: Fig. S2. The immunofluorescent staining in 3 and 4-month-old human embryonic TMJC. a The expression of COL10A1, MMP13 and RUNX2 in each cluster of human embryonic TMJC. b The expression of FGFBP2 and SCIN in each cluster of human embryonic TMJC. c Immunofluorescence staining of FGFBP2 and SCIN in 3 and 4-month-old human embryonic TMJC, Scale bar = 1 mm and 50 µm. [file 13578_2023_1069_MOESM2_ESM.pdf]

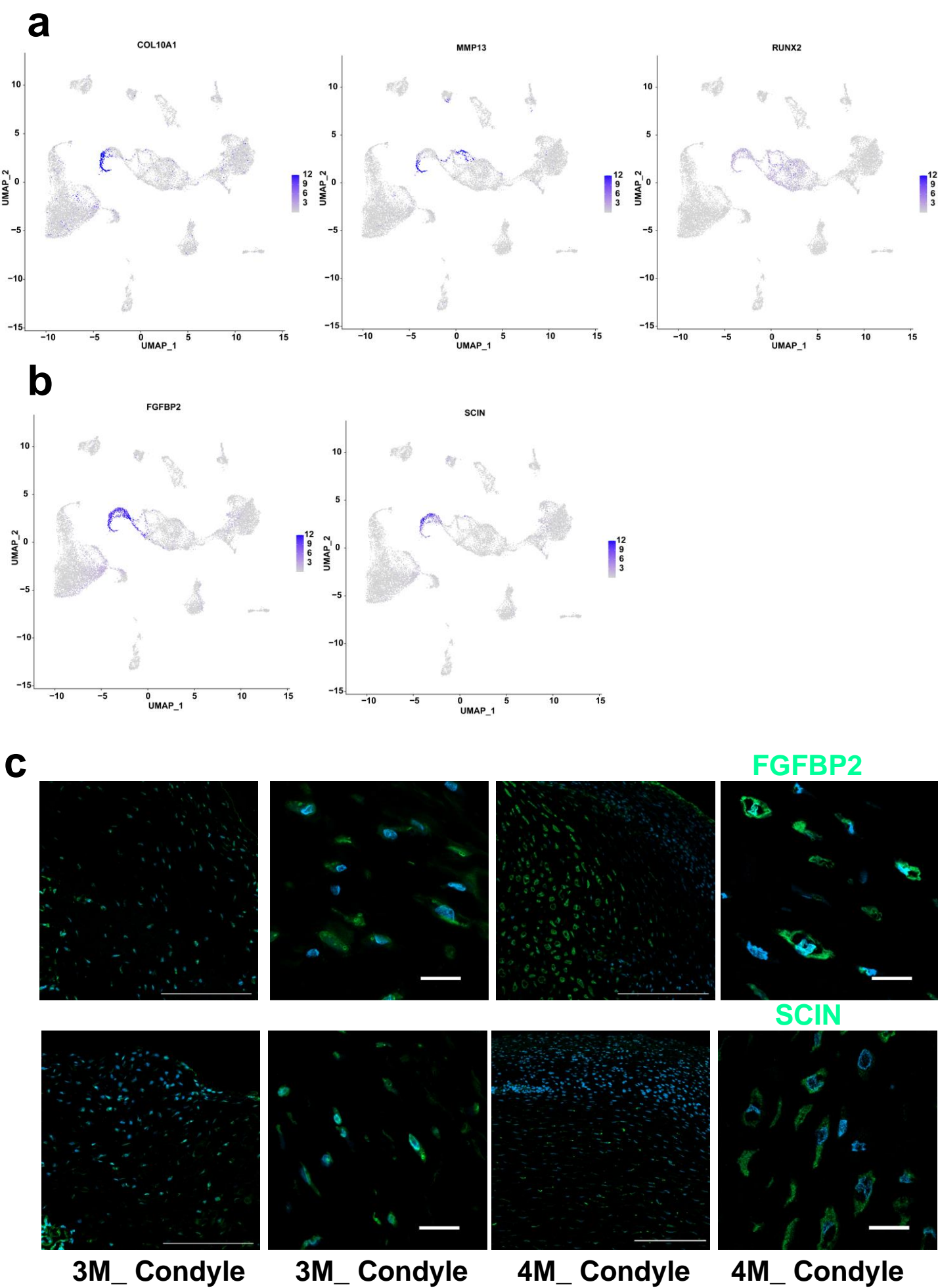

**Fig. S2**

Supplement: Supplementary file 3 — Additional file 3: Fig. S3. The differentiation relationship among human embryonic TMJC cells. a The differentiation relationship among 15 TMJC cell clusters based on Monocle3 analysis. b The RNA velocity analysis of 15 TMJC cell clusters. c The pseudotime analysis among MSCs, TSCs, tenocytes, hypertrophic chondrocytes and osteoblasts of the 3 and 4-month-old human embryonic TMJC. d The RNA velocity analysis among MSCs, TSCs, tenocytes, hypertrophic chondrocytes and osteoblasts of the 3 and 4-month-old human embryonic TMJC. [file 13578_2023_1069_MOESM3_ESM.pdf]

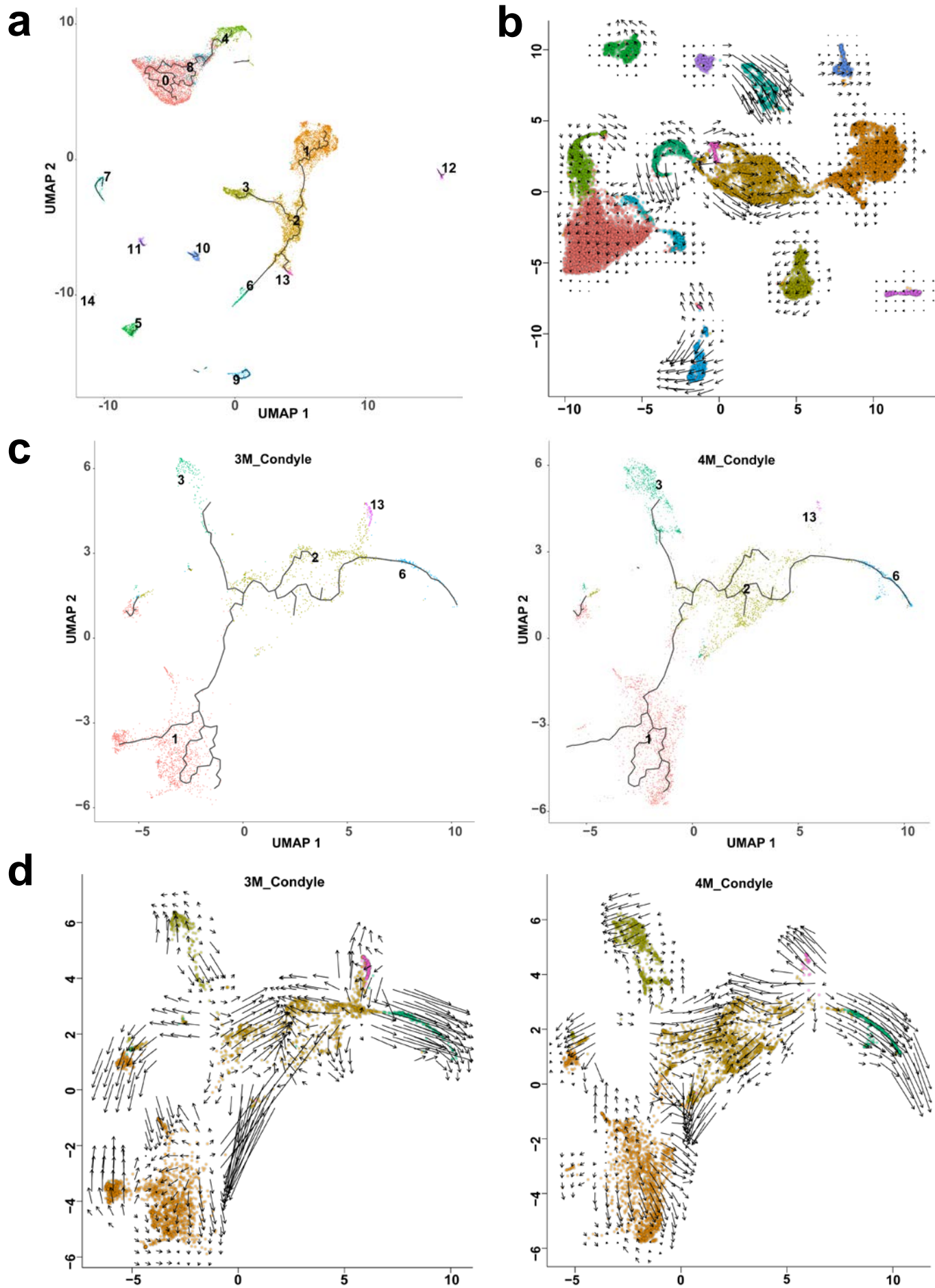

Supplement: Supplementary file 4 — Additional file 4: Fig. S4. Cell Chat analysis of human embryonic TMJC cell clusters. a The interaction strength analysis of ligand-receptor pairs in 15 cell clusters. b FGF signaling pathway networks among 15 cell clusters. c The expression analysis of FGFR1 among 15 cell clusters. d FGF7-FGFR1 signaling pathway networks among 15 cell clusters. e The analysis of outgoing signaling patterns and incoming signaling patterns among 15 cell clusters. [file 13578_2023_1069_MOESM4_ESM.pdf]
