# Supplementary material for: Single-cell RNA sequencing analysis of the temporomandibular joint condyle in 3 and 4-month-old human embryos
Source: Cell Biosci. 2023 Jul 19;13:130. doi: 10.1186/s13578-023-01069-5 (PMC10357633; doi:10.1186/s13578-023-01069-5)
Supplement: Supplementary file 6 — Additional file 6. [file 13578_2023_1069_MOESM6_ESM.docx]

| Clusters | Marker genes |
| --- | --- |
| 0 Satellite cells | PAX7 (Zammit et al., 2006), MYF5 (Zammit, 2017) |
| 1 Mesenchymal stem cells | NRK (Niu et al., 2019), PDGFRA (Uezumi et al., 2014) |
| 3 Tenocytes | KERA (Mueller, Tew, Vasieva, Clegg, & Canty-Laird, 2016), TNMD (Qi et al., 2012) |
| 4 Myoblast | MYOG (Faralli & Dilworth, 2012), KLHL41 (Jirka, Pak, Grosgogeat, Marchetii, & Gupta, 2019) |
| 5 Endothelial cells | CDH5 (Sauteur et al., 2014), PECAM1 (Privratsky & Newman, 2014) |
| 6 Hypertrophic chondrocytes | LOXL4 (Iftikhar et al., 2011), COL10A1 (Gu et al., 2014) |
| 7 Erythrocytes | HBA1 (L. N. Zhu et al., 2020), HBA2 (L. N. Zhu et al., 2020) |
| 8 Proliferating cells | MKI67 (Hou et al., 2011), TOP2A (An et al., 2018) |
| 9 Leukocytes | PTPRC (Abdollahi, Kohn, & Borset, 2021), CXCL8 (Mukaida, 2003) |
| 10 Pericytes | RGS5 (Cho, Kozasa, Bondjers, Betsholtz, & Kehrl, 2003), ABCC9 (Vanlandewijck et al., 2018) |
| 11[Chondrocyte](javascript:;) | CYTL1 (Tomczak et al., 2017), ITGA10 (G. Zhu et al., 2015) |
| 12 Schwann cells | PLP1 (Garbern et al. 1997), MPZ (Hasse et al. 2002)/(Hasse et al. 2002) |
| 13 Osteoblasts | IBSP (Komori & Biosciences, 2010), ALPL (Techaniyom, Tanurat, & Sirivisoot, 2020) |
| 14 Osteoclasts | RGS10 (Yang and Li 2007), MMP9 (Liu et al., 2016) |

Table2 Marker genes for annotation of TMJC cell clusters

Abdollahi, P., Kohn, M., & Borset, M. (2021). Protein tyrosine phosphatases in multiple myeloma. *Cancer Letters, 501*, 105-113. doi:10.1016/j.canlet.2020.11.042

An, X., Xu, F., Luo, R., Zheng, Q., Lu, J., Yang, Y., . . . Wang, S. (2018). The prognostic significance of topoisomerase II alpha protein in early stage luminal breast cancer. *BMC Cancer, 18*(1), 331. doi:10.1186/s12885-018-4170-7

Cho, H., Kozasa, T., Bondjers, C., Betsholtz, C., & Kehrl, J. H. (2003). Pericyte-specific expression of Rgs5: implications for PDGF and EDG receptor signaling during vascular maturation. *FASEB J, 17*(3), 440-442. doi:10.1096/fj.02-0340fje

Faralli, H., & Dilworth, F. J. (2012). Turning on myogenin in muscle: a paradigm for understanding mechanisms of tissue-specific gene expression. *Comp Funct Genomics, 2012*, 836374. doi:10.1155/2012/836374

Gu, J., Lu, Y., Li, F., Qiao, L., Wang, Q., Li, N., . . . Zheng, Q. (2014). Identification and characterization of the novel Col10a1 regulatory mechanism during chondrocyte hypertrophic differentiation. *Cell Death Dis, 5*, e1469. doi:10.1038/cddis.2014.444

Hou, Y. Y., Cao, W. W., Li, L., Li, S. P., Liu, T., Wan, H. Y., . . . Tang, H. (2011). MicroRNA-519d targets MKi67 and suppresses cell growth in the hepatocellular carcinoma cell line QGY-7703. *Cancer Letters, 307*(2), 182-190. doi:10.1016/j.canlet.2011.04.002

Iftikhar, M., Hurtado, P., Bais, M. V., Wigner, N., Stephens, D. N., Gerstenfeld, L. C., & Trackman, P. C. (2011). Lysyl oxidase-like-2 (LOXL2) is a major isoform in chondrocytes and is critically required for differentiation. *J Biol Chem, 286*(2), 909-918. doi:10.1074/jbc.M110.155622

Jirka, C., Pak, J. H., Grosgogeat, C. A., Marchetii, M. M., & Gupta, V. A. (2019). Dysregulation of NRAP degradation by KLHL41 contributes to pathophysiology in nemaline myopathy. *Hum Mol Genet, 28*(15), 2549-2560. doi:10.1093/hmg/ddz078

Komori, & Biosciences, T. J. J. o. O. (2010). Regulation of Osteoblast and Odontoblast Differentiation by RUNX2. *52*(1), 22-25.

Liu, B., Cui, J., Sun, J., Li, J., Han, X., Guo, J., . . . Li, M. (2016). Immunolocalization of MMP9 and MMP2 in osteolytic metastasis originating from MDA-MB-231 human breast cancer cells. *Mol Med Rep, 14*(2), 1099-1106. doi:10.3892/mmr.2016.5374

Mueller, A. J., Tew, S. R., Vasieva, O., Clegg, P. D., & Canty-Laird, E. G. (2016). A systems biology approach to defining regulatory mechanisms for cartilage and tendon cell phenotypes. *Sci Rep, 6*, 33956. doi:10.1038/srep33956

Mukaida, N. (2003). Pathophysiological roles of interleukin-8/CXCL8 in pulmonary diseases. *American Journal of Physiology-Lung Cellular and Molecular Physiology, 284*(4), L566-L577. doi:10.1152/ajplung.00233.2002

Niu, X., Li, J., Zhao, X., Wang, Q., Wang, G., Hou, R., . . . Zhang, K. (2019). Dermal mesenchymal stem cells: a resource of migration-associated function in psoriasis? *Stem Cell Res Ther, 10*(1), 54. doi:10.1186/s13287-019-1159-3

Privratsky, J. R., & Newman, P. J. (2014). PECAM-1: regulator of endothelial junctional integrity. *Cell Tissue Res, 355*(3), 607-619. doi:10.1007/s00441-013-1779-3

Qi, J., Dmochowski, J. M., Banes, A. N., Tsuzaki, M., Bynum, D., Patterson, M., . . . Banes, A. J. (2012). Differential expression and cellular localization of novel isoforms of the tendon biomarker tenomodulin. *J Appl Physiol (1985), 113*(6), 861-871. doi:10.1152/japplphysiol.00198.2012

Sauteur, L., Krudewig, A., Herwig, L., Ehrenfeuchter, N., Lenard, A., Affolter, M., & Belting, H. G. (2014). Cdh5/VE-cadherin promotes endothelial cell interface elongation via cortical actin polymerization during angiogenic sprouting. *Cell Rep, 9*(2), 504-513. doi:10.1016/j.celrep.2014.09.024

Techaniyom, P., Tanurat, P., & Sirivisoot, S. (2020). Osteoblast differentiation and gene expression analysis on anodized titanium samples coated with graphene oxide. *Applied Surface Science, 526*. doi:ARTN 146646

10.1016/j.apsusc.2020.146646

Tomczak, A., Singh, K., Gittis, A. G., Lee, J., Garboczi, D. N., & Murphy, P. M. (2017). Biochemical and biophysical characterization of cytokine-like protein 1 (CYTL1). *Cytokine, 96*, 238-246. doi:10.1016/j.cyto.2017.04.023

Uezumi, A., Fukada, S., Yamamoto, N., Ikemoto-Uezumi, M., Nakatani, M., Morita, M., . . . Tsuchida, K. (2014). Identification and characterization of PDGFRalpha+ mesenchymal progenitors in human skeletal muscle. *Cell Death Dis, 5*, e1186. doi:10.1038/cddis.2014.161

Vanlandewijck, M., He, L. Q., Mae, M. A. A., Andrae, J., Ando, K., Del Gaudio, F., . . . Betsholtz, C. (2018). A molecular atlas of cell types and zonation in the brain vasculature. *Nature, 554*(7693), 475-+. doi:10.1038/nature25739

Zammit, P. S. (2017). Function of the myogenic regulatory factors Myf5, MyoD, Myogenin and MRF4 in skeletal muscle, satellite cells and regenerative myogenesis. *Semin Cell Dev Biol, 72*, 19-32. doi:10.1016/j.semcdb.2017.11.011

Zammit, P. S., Relaix, F., Nagata, Y., Ruiz, A. P., Collins, C. A., Partridge, T. A., & Beauchamp, J. R. (2006). Pax7 and myogenic progression in skeletal muscle satellite cells. *J Cell Sci, 119*(Pt 9), 1824-1832. doi:10.1242/jcs.02908

Zhu, G., Mayer-Wagner, S., Schroder, C., Woiczinski, M., Blum, H., Lavagi, I., . . . Muller, P. E. (2015). Comparing effects of perfusion and hydrostatic pressure on gene profiles of human chondrocyte. *J Biotechnol, 210*, 59-65. doi:10.1016/j.jbiotec.2015.06.409

Zhu, L. N., Yang, P. H., Zhao, Y. Z., Zhuang, Z. K., Wang, Z. F., Son, R., . . . Liu, W. J. (2020). Single-Cell Sequencing of Peripheral Mononuclear Cells Reveals Distinct Immune Response Landscapes of COVID-19 and Influenza Patients. *Immunity, 53*(3), 685-+. doi:10.1016/j.immuni.2020.07.009
